# Roles of sphingosine-1-phosphate signaling in cancer

**DOI:** 10.1186/s12935-019-1014-8

**Published:** 2019-11-14

**Authors:** Peng Wang, Yonghui Yuan, Wenda Lin, Hongshan Zhong, Ke Xu, Xun Qi

**Affiliations:** 1 Key Laboratory of Diagnostic Imaging and Interventional Radiology of Liaoning Province, Department of Radiology, The First Affiliated Hospital of China Medical University, Shenyang, 110001 Liaoning China; 2 Research and Academic Department, Cancer Hospital of China Medical University Liaoning Cancer Hospital & Institute, Shenyang, 110042 Liaoning China

**Keywords:** Cancer progression, Sphingosine-1-phosphate, Sphingosine 1-phosphate receptors, Therapeutic target, Signaling modulators

## Abstract

The potent pleiotropic lipid mediator sphingosine-1-phosphate (S1P) participates in numerous cellular processes, including angiogenesis and cell survival, proliferation, and migration. It is formed by one of two sphingosine kinases (SphKs), SphK1 and SphK2. These enzymes largely exert their various biological and pathophysiological actions through one of five G protein-coupled receptors (S1PR1–5), with receptor activation setting in motion various signaling cascades. Considerable evidence has been accumulated on S1P signaling and its pathogenic roles in diseases, as well as on novel modulators of S1P signaling, such as SphK inhibitors and S1P agonists and antagonists. S1P and ceramide, composed of sphingosine and a fatty acid, are reciprocal cell fate regulators, and S1P signaling plays essential roles in several diseases, including inflammation, cancer, and autoimmune disorders. Thus, targeting of S1P signaling may be one way to block the pathogenesis and may be a therapeutic target in these conditions. Increasingly strong evidence indicates a role for the S1P signaling pathway in the progression of cancer and its effects. In the present review, we discuss recent progress in our understanding of S1P and its related proteins in cancer progression. Also described is the therapeutic potential of S1P receptors and their downstream signaling cascades as targets for cancer treatment.

## Background

The vast sphingolipid family includes two prominent bioactive lipids, ceramide and sphingosine-1-phosphate (S1P). The latter, which is membrane derived, is produced through ceramidase-catalyzed conversion of ceramide to sphingosine and its subsequent phosphorylation by sphingosine kinases (SphKs) [1]. Export of intracellularly produced S1P requires the action of some transporters, particularly ATP-binding cassette (ABC) C1, ABCG2, and spinster 2 (Spns2) [2, 3]. S1P exerts a multitude of effects on cell physiology and pathology through five S1P receptors (S1PRs) [4]. These receptors are coupled to distinct G protein α subtypes, including Gq, Gi, and G12/13 [5]. Activation of the S1PRs and the consequent stimulation of the associated G protein regulates distinct signaling pathways, including AKT and ERK1/2 [6]. Together, these cascades mediate S1P signaling, which is involved in numerous diseases, including cancer, atherosclerosis, kidney disease, and immunological disorders [7–9]. In recent years, considerable progress has been made in elucidating the role of S1P signaling in cancer. In particular, Besim Ogretmen highlighted the role of S1PR-dependent and -independent signaling in cancer cell growth, tumor metastasis, and drug resistance [10]. In addition, some studies have found that S1PR2 plays a dual role in the progression of cancer by modulating a diverse range of downstream second messengers [11, 12]. Therefore, targeting of S1P signaling is likely to be a novel therapeutic strategy for cancer.

In this review, we discuss S1P signaling and its involvement in cancer progression. We additionally describe the potential value of S1P signaling pathway targeting in cancer therapy.

## S1P, a bioactive lipid mediator

More than 25 years ago, Sarah Spiegel discovered that S1P is a biologically active lipid mediator that is abundant in plasma, regulates many physiological processes, and acts as a signaling molecule in cells [13, 14]. Since then, more and more researchers have explored S1P synthesis, degradation, and receptors, all of which collaborate to regulate S1P expression and signaling inside and outside cells [15]. Collectively, these findings prove that S1P acts as a signaling molecule in cells. The validation of S1P and its related molecules has boosted lipid research [16].

## S1P biosynthesis and homeostasis

S1P is derived from intracellular ceramide. Ceramide is first converted into sphingosine by the enzyme ceramidase, with sphingosine subsequently phosphorylated by SphK to produce S1P [1]. Thus far, two SphK isotypes—SphK1 and SphK2—have been identified [17]. The former is present in nearly every cell type [18] and is localized to the cytosol close to the cell membrane, where it participates in the transport of S1P via “inside-out” signaling [19]. “Inside-out” signaling refers to the process through which S1P produced inside cells is exported by transporters and signals through extracellular S1PRs [2, 15], which will be described later in this review. It is through this process that we can find a large number of S1Ps in some cancers, such as breast, gastric, and pancreatic [20–23]. Because SphK2 has mainly been reported in the nuclei and mitochondria, S1P produced by SphK2 may be not as effective as S1P extracellularly produced by SphK1 [24]. Furthermore, although S1P is structurally no different whether produced by SphK1 or SphK2, it exerts different functions in the body according to where it has been produced [15]. Thus, S1P in the nuclei acts as a histone deacetylase inhibitor and epigenetically regulates gene transcription [24, 25]. SphK2 is highly expressed and plays complex roles in highly diverse organs, including the kidney, liver, and brain. For example, S1P in the nucleus and mitochondrion controls the transcription of genes critical to epigenetics [26, 27]. Moreover, SphK2 may participate in mast cell function, with SphK2 inhibition ameliorating immunosuppression-related disorders, such as chronic infections and/or cancers [28–30].

Despite its role as a pleiotropic lipid mediator, S1P cannot freely pass through plasma membranes to the extracellular space due to the presence of a polar head group. S1P export thus requires the involvement of a transporter [15], and several such proteins have been identified in recent years [19, 31]. Indeed, some ABC transporters have been found to export S1P, including ABCC1 [2, 31]. The S1P transporter function of ABCC1 was first identified in mast cells [32]. ABCC1 and the related transporter ABCG2 transport S1P out of estrogen receptor-positive breast cancer cells in response to the nongenomic actions of estrogen [33]. More recently, Spns2, a member of the major facilitator superfamily that lacks the typical ATP-binding motif [34, 35], was determined by two independent groups to be an S1P transporter [3, 35]. Both research groups, which identified the S1P-transporting function of Spns2 in zebrafish, showed that a mutation in *Spns2* led to cardia bifida (duplicated hearts). The phenotype could be rescued using exogenous S1P [33, 36].

S1P is present in higher concentrations in blood and lymph than in tissue [37]. In addition, S1P-degrading enzymes are more active in tissue, where they play a major role in limiting the levels of S1P. Two enzymes reduce the level of S1P: S1P lyase and S1P phosphatase [38]. S1P lyase irreversibly decomposes S1P by cleaving its C2–C3 bond [39]. Some studies have shown that S1P lyase expression is significantly downregulated in human colon cancer tissues versus normal adjacent tissues [40, 41], an indicator of the importance of low S1P levels. As part of a recycling pathway, S1P phosphatase hydrolyzes the phosphate group from S1P to produce sphingosine, which is then converted by ceramide synthase to ceramide [42].

**Fig. 1:**
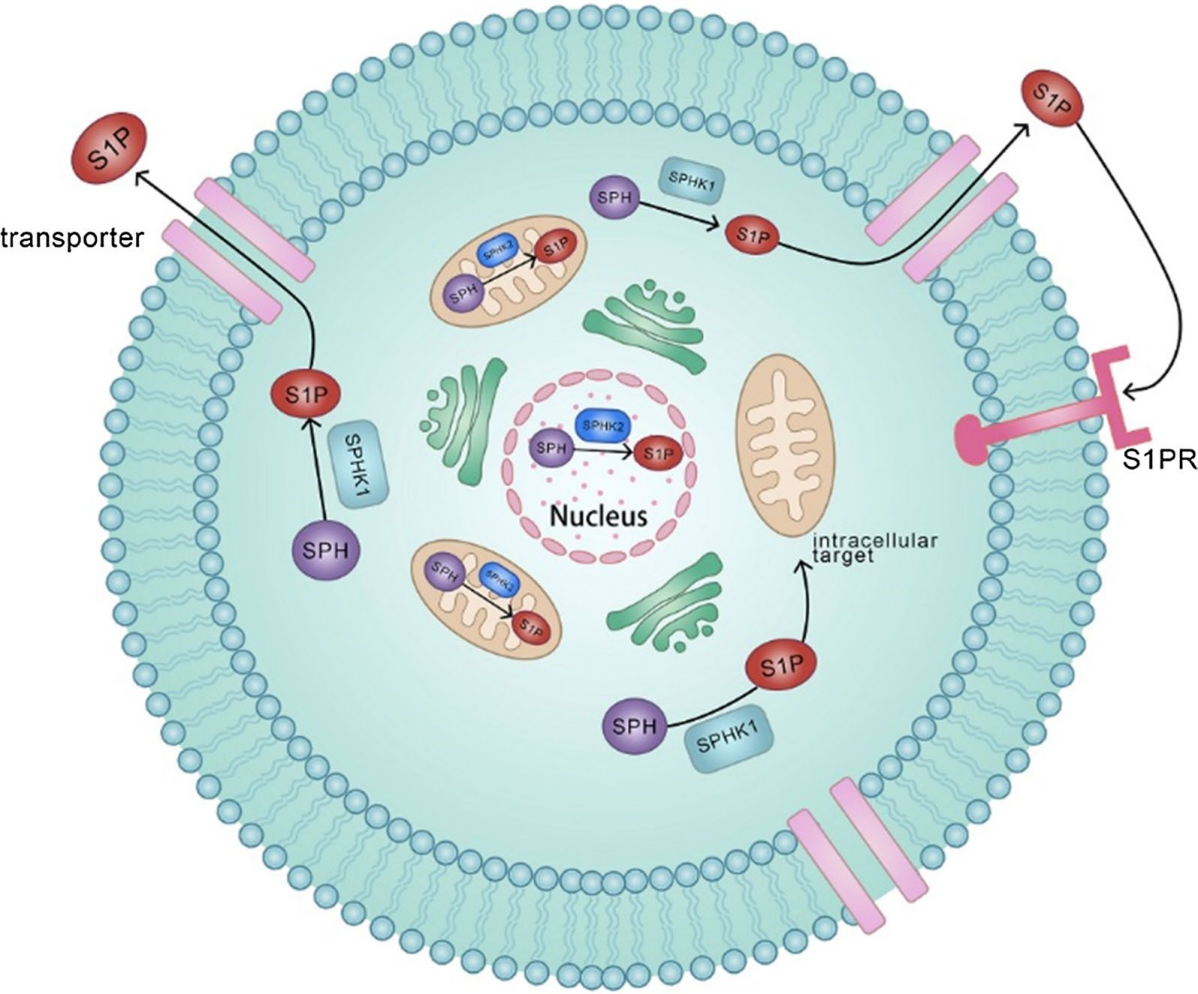
Biosynthesis of S1P. S1P is generated from sphingosine (SPH) by two sphingosine kinases (SphK1 and SphK2) in the catabolic pathway. SphK1 mainly exists in the cytosol, but SphK2 exists in the nuclei and mitochondria. S1P produced by SphK1 is exported to the extracellular space, where it exerts various functions associated with cancer via S1P receptor (S1PR). S1P produced by SphK2 is thought to play important roles in intracellular functions

Taken together, SphK, S1P transporter, and its degrading enzymes all regulate S1P gradation and signaling (Fig. 1), which control normal physiological function and may play a role in cancer progression.

## S1P receptors and agonists/antagonists

S1P, whether produced by SphK1 or SphK2, owes almost all of its bioactive pleiotropic effects on cell survival, migration, angiogenesis, and lymphangiogenesis and immune cell recruitment, all processes that may be involved in cancer, to S1PR1–5, which are S1P-specific G protein-coupled receptors (GPCRs) [4, 43]. These five receptors are canonical members of the rhodopsin subfamily of GPCRs (class A). Their characteristic features comprise an intracellular C terminus, seven helical transmembrane domains, and a 30 to 50 residue extracellular N terminus. Deorphanization work has recently determined that S1PRs, similar to a larger-than-expected number of GPCRs (~ 40 so far), are selectively activated by bioactive lipids, such as leukotrienes, prostaglandins, free fatty acids, endocannabinoids, and phospholipids (including lysophosphatidic acid [LPA] and lysophosphatidylserine) [44, 45]. Closely related to the S1PRs are LPA (LPA1–3) receptors [15, 46], which bind a lipid with a similar structure to S1P. The receptors in this subfamily show considerable sequence homology to each other and, although closely related to endocannabinoid receptors, are divergent from the other lipid-activated GPCRs.

Knowledge of the structure and mechanism of S1PRs may help to shed light on the diseases in which they participate, including atherosclerosis, cancer [7, 40, 47–49], diabetes [50], congenital disorders [36], kidney diseases [8], and immunological diseases [9]. Recent efforts have yielded diverse compounds, both agonists and antagonists and with varying degrees of selectivity, that affect S1PRs [51] (Table 1). Notably, major breakthroughs have been made in immune diseases, although the vast majority of compound research is still in the preclinical stage. For example, fingolimod (FTY720; trade name Gilenya) was approved in 2010 by the American Food and Drugs Administration for the treatment of multiple sclerosis [52, 53]. This compound is an S1P agonist that binds to S1PR1, -3, -4, and -5 to stimulate their internalization and degradation, leading to their downregulation. In addition, it can directly inhibit SphK1 activity. Although it has been used clinically, its efficacy is poor. A randomized, double-blind, placebo-controlled trial of oral fingolimod in primary progressive multiple sclerosis indicated that fingolimod, despite its anti-inflammatory activity, failed to slow the progression of primary progressive multiple sclerosis [54]. Shortly afterward, Chitnis et al. [55] proposed that longer studies be performed to elucidate fingolimod safety and durability in pediatric patients with multiple sclerosis. In contrast, fingolimod potently prevents the Ca^2+^ mobilization and migration induced by S1P in vascular endothelial cells and inhibits tumor vascularization and growth in some cancer models, such as Lewis lung carcinoma [56], colon cancer [57], hepatocellular carcinoma [58], and prostate cancer [59]. Furthermore, Beider et al. [60] identified cross-talk between the S1P and CXCR4 pathways in multiple myeloma cells. They subsequently demonstrated that fingolimod effectively reduced tumor burden in the bone marrow of multiple myeloma-bearing mice. Besides fingolimod, the remaining S1P agonists, such as siponimod, SEW2817, AUY954, ponesimod, and KRP-203, mainly target S1PR1, but their roles and mechanisms still need further study.

**Table 1:** The roles played by S1P agonists and antagonists

S1P modulators	Targets	Cancer models	Functions
S1P agonists			
Fingolimod (FTY720)	S1PR1, S1PR3, S1PR4, S1PR5	Lewis lung carcinoma [56], colon cancer [57], hepatocellular carcinoma [58], prostate cancer [59], multiple myeloma [60]	Suppressing angiogenesis [56], suppressing tumor growth and metastasis [57, 59], suppressing aggressiveness [60], inducing apoptosis [58]
AUY954	S1PR1	Unknow yet	
KRP-203	S1PR1	Unknow yet	
SEW2817	S1PR1	Unknow yet	
Siponimod	S1PR1	Unknow yet	
Ponesimod	S1PR1	Unknow yet	
S1P antagonists			
TY52156	S1PR3	Unknow yet	Promoting tumor aggressiveness [67]
JTE-013	S1PR2, S1PR3, S1PR4	Unknow yet	
CYM-5478	S1PR2	Oral squamous cell carcinoma [67]	

As discussed above, and as an example of “functional antagonism”, binding of fingolimod to its receptors triggers their internalization and degradation. Therefore, S1P antagonists, by directly competing with S1P, are likely to obstruct downstream pathways. Evidence supporting this hypothesis was provided by knockdown of S1PR1 in T cells [61]. Unlike S1P agonists, antagonists of S1P can target different types of S1PRs. Murakami et al. [62] found that TY52156, as an antagonist of S1PR3, could directly inhibit S1P-mediated vascular contraction by activating calcium and Rho in vascular smooth muscle cells. JTE-013 is a controversial S1PR antagonist, with preclinical studies considering it an S1P2-selective antagonist [63]. However, recent studies suggested that submicromolar concentrations of JTE-013 antagonize S1P4, with higher concentrations affecting S1P3 [64, 65]. On the other hand, CYM-5478 appears to be a highly selective agonist of S1P2 [66]. Research work used CYM-5478 to establish in vitro and in vivo roles for S1P2, in cancer cells [67] and renal ischemia–reperfusion injury [68], respectively. Similarly, initial work has reported both an antagonist and an agonist that selectively act on S1P3. Collectively, these findings indicate the major advances in S1P antagonist development. Furthermore, compared with S1PR antagonist, these compounds remain to be examined in cancer models. Accordingly, further work is required to determine the antitumor properties of S1P antagonists.

## Specific roles for the individual receptor subtypes in cancer

**Fig. 2:**
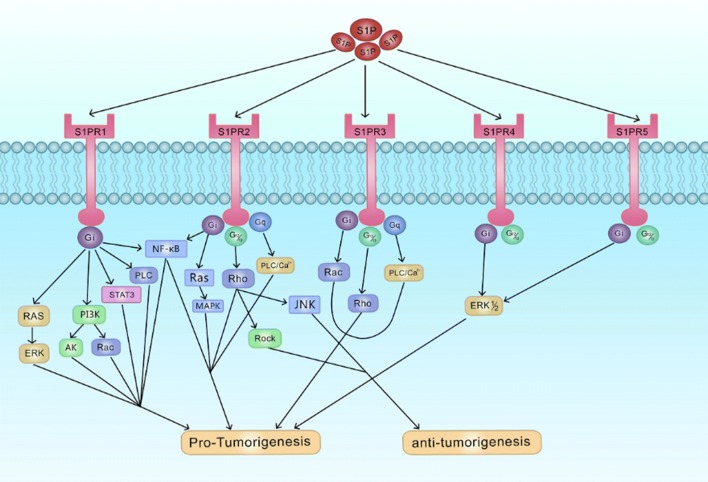
S1P receptor signaling. S1P exported to the extracellular space binds to the receptors and activates the receptor-bound G-proteins (Gi, Gq, G12/13). The activated G-proteins subsequently trigger the downstream pathways, which plays a role in growth, proliferation, migration and invasion of cancer

Most studies investigating the involvement of S1P signaling in cancer have typically relied on manipulating S1P metabolism or using nonselective receptor ligands. Nonetheless, genetic manipulation and correlation analyses have shed some light on the varied, and even conflicting, functions of different receptor subtypes. Briefly, the signaling pathways activated by S1P through S1PR1–5 signaling play specific roles in regulating the proliferation, migration, and/or invasion of cancer cells in a context-dependent manner (Fig. 2).

### The role of S1PR1 in cancer

S1PR1, when specifically bound to Gi, activates numerous signaling pathways, such as Ras/ERK, NLRP3/IL-1β, PI3K/AKT, PI3K/Rac, STAT3, and PLC, and is largely regarded as a protumorigenic factor, promoting migration, invasion, proliferation, and neovascularization in various kinds of cancer [69]. RNA interference experiments have shown that S1PR1 is required in vivo for tumor angiogenesis [70]. In breast cancer, the SphK1/S1P/S1PR1 axis is critically involved in regulating numerous cellular processes, including cell growth, survival, invasion, vascular integrity, immune cell trafficking, angiogenesis, and cytokine and chemokine production [19, 21, 71–73]. Therefore, the question is whether we can decrease tumor viability and growth by inhibiting S1PR1 expression. The answer is, unfortunately, no. Liu et al. [74] showed that S1PR1 can accelerate VE-cadherin phosphorylation (Y731) by activating RhoA, leading to increased endothelium-dependent vessel and reduced vasculogenic mimicry in breast cancer, and the low expression of S1PR1 is in line with the vasculogenic mimicry and poor prognosis in breast cancer patients. In the mouse B16 tumor model, activated S1PR1 can accelerate tumor progression by activating STAT3 and upregulating IL-6 [75], and this relationship between S1PR1 and STAT3 has been shown to contribute to chronic intestinal inflammation and colitis-associated colon cancer [57]. In Wilms tumor (also called nephroblastoma), S1PR1 stimulates migration and invasion through PI3K and the Rac pathway, which has promigratory properties [76]. S1PR1 overexpression, which contributes to the expression and activity of urokinase plasminogen activator (uPA), significantly expedites invasion in glioblastoma [77]. In addition, S1PR1 can also activate ERK to enhance cell survival [78] and promote cell migration in fibrosarcoma [79] and Hodgkin lymphoma [80]. A recent study illustrated that the microRNA302-367-ERK1/2-KLF2-S1PR1 pathway can restrict angiogenesis and stabilize vascular cells, which means that the ERK1/2-KLF2-S1PR1 pathway may promote tumor growth by stimulating angiogenesis [81].

### The role of S1PR2 in cancer

S1PR2, in contrast to S1PR1, can bind to multiple Gα proteins, including Gi, G12/13, and Gq. Therefore, S1PR2 can play multiple functions in the progression of cancer by affecting a diverse range of downstream second messengers.

The protumorigenic potential of S1P2 activation has been indicated in some recent studies. Cholangiocarcinoma has been closely linked to chronic cholestasis and significantly increased levels of bile acids, both primary and conjugated. Liu et al. [82] suggested that conjugated bile acids can activate ERK1/-2 and AKT signaling pathways through S1PR2 in rodent hepatocytes. Furthermore, their subsequent findings indicated that S1PR2 is vital for conjugated bile acid-mediated cholangiocarcinoma cell growth and invasion. In Wilms tumor, S1PR2 was found to contribute to tumor growth and angiogenesis by stimulating the expression of cyclooxygenase 2 (COX-2) [11]. In glioma cells, Young and Van Brocklyn [83] found that overexpression of S1PR2 significantly increased the invasiveness of U-118 MG cells, despite decreasing cell motility. A more detailed study, which included in vivo and in vitro experiments related to pancreatic cancer, demonstrated that S1P activates pancreatic stellate cells to produce matrix metalloproteinase-9 (MMP-9) through an S1P2-, c-Abl-, and NF-κB-dependent pathway. This series of reactions ultimately stimulates pancreatic cancer cell migration and invasion [84]. An in vivo allograft study revealed that communication from SphK/S1P signaling of host cells with S1P2 expressed by allografted bladder cancer cells facilitates lung cancer metastasis [85]. In hepatocellular carcinoma, Cheng et al. [86] showed that S1P plays a role in stimulating hepatocellular carcinoma cell proliferation by upregulating connective tissue growth factor (CTGF) expression through S1P2-mediated Yes-associated protein (YAP) activation. Besides its tumorigenicity, S1PR2 may increase resistance to imatinib and nilotinib in chronic myeloid leukemia patients by inhibiting the activity of protein phosphatase 2A and thereby stabilizing Bcr-Abl1 protein [87]. In addition, non-neoplastic work has shown that activation of S1P2 can increase cell viability and reduce cisplatin-mediated cell death by decreasing reactive oxygen species (ROS) [66].

Surprisingly, S1PR2 also plays an important role in suppressing cancer. S1PR1–5 and S1P metabolic enzymes are found in three different human glioblastoma cell lines and S1P has wide-ranging effects on glioblastoma cell migration [88]. Moreover, Lepley et al. [89], after using FTY720-P, one of the S1P analogues, to bind all S1P receptors except S1PR2, found that migration was not suppressed. However, overexpression of S1P2 inhibited migration, and their further work illustrated that S1P_2_ has a suppressive effect on glioblastoma cell migration, although this did not correlate with proliferation. In thyroid cancer, Asghar et al. [90] demonstrated that S1P inhibits the invasive properties of C643 thyroid cancer cells by activating S1P2 and the Rho-ROCK pathway. In Wilms tumor, the effect of S1PR2 is different from that discussed above, and S1PR2 appears to stimulate the Rho/Rho kinase/JNK pathway and thereby block cell proliferation by inducing the immediate expression of CTGF [12]. Moreover, some studies indirectly illustrated the antitumorigenic activity of S1PR2. For example, disruption of the gene expression of S1P receptors for S1P2 leads to diffuse large B-cell lymphoma (DLBCL) formation in S1P_2_ knockout mice [91]. Similarly, Stelling et al. [92] manifested the suppressive function of the TGF-β/TGF-βR2/SMAD1/S1PR2 axis in DLBCL and showed that DLBCL cells have evolved to be able to inactivate the pathway at the SMAD1 expression level. The 14-3-3ζ protein may promote the invasion of esophageal squamous cell carcinoma by repressing S1PR2 expression through NF-κB signaling [93].

### The role of S1PR3 in cancer

S1PR3, coupled to Gi, Gq, and G12/13, has a close correspondence with human breast cancer. Not only is it the most highly expressed S1PR in human breast cancer cell lines [94], but also its activation may promote tumor progression and reduce overall survival in patients with breast cancer [95–97]. Consistently associated with Epstein-Barr virus infection, the pathogenesis of nasopharyngeal carcinoma has also recently been linked to S1P/S1PR3 signaling. S1PR3 mRNA is overexpressed in Epstein–Barr virus-positive nasopharyngeal carcinoma patient-derived xenografts and some primary nasopharyngeal carcinoma tissues and its knockdown blocks both AKT activation and the migration of nasopharyngeal carcinoma cells induced by S1P [98]. Because S1PR3 levels are elevated in a series of human lung adenocarcinoma cell lines, Zhao et al. [99] found that TGF-β-stimulated S1PR3 upregulation boosts the proliferation of human lung adenocarcinoma cells in mice through the TGF-β/SMAD3 pathway; in contrast, knockdown of S1PR3 markedly blocks tumor growth and lung metastasis. Earlier studies showed that S1P/S1PR3 signaling increases the expression of endothelial growth factor receptors (EGFR) in lung adenocarcinoma cells through the Rho kinase pathway and enhances EGF-stimulated cell proliferation, cell invasion, and colony formation [100]. Like S1P1, S1P3 contributes to angiogenesis, although less strongly [101]. The S1P/S1PR3 axis is involved in promoting proliferation, inhibiting apoptosis, and accelerating aerobic glycolysis in osteosarcoma cells via the YAP/c-MYC/PGAM1 pathway [102].

### The role of S1PR4 and S1PR5 in cancer

Compared with S1PR1–3, S1PR4 and S1PR5 have a restricted distribution and less clear functions. Current research indicates that S1PR4 and S1PR5 couple to Gi and G12/13 [103, 104]. In breast cancer, S1P/S1PR4 signaling can activate the Erk1/2 and human epidermal growth factor receptor-2 (HER2) pathways and increase tumor aggressiveness [105, 106]. It is also correlated with worse outcomes and shorter survival in estrogen receptor-negative breast cancer [105]. Moreover, by activating S1PR4 signaling, SPHK2-generated S1P prevents the nuclear translocation of S1PR2 and boosts the proliferation of estrogen receptor-negative breast cancer cells [107]. Nonetheless, further work is required to clarify how S1PR4 regulates the nuclear translocation of S1PR2, as well as how nuclear S1PR2 inhibits the growth of estrogen receptor-negative breast cancer cells. S1P5 boosts survival in prostate cancer by inducing autophagy under serum-deprived conditions [108]. However, S1P5 is expressed at a lower level in esophageal squamous cell carcinoma than in normal esophageal mucosal epithelium [109]. In addition, S1PR4 and S1PR5 play important roles in inflammation, which is also closely related to the progression of some cancers, such as colon cancer [110].

## The therapeutic potential of S1P signaling in cancer

As reviewed here, numerous studies have demonstrated that S1P signaling is closely associated with cancer progression. Moreover, growing evidence indicates S1P signaling as a potential therapeutic target. Thus far, focusing on S1P signaling as a promising therapeutic target, there are two main research strategies: one is to reduce the levels of S1P itself, the other is to agonize/antagonize S1P receptors. Two drug candidates have been clinically used to reduce S1P levels in the oncology field. A phase II trial of the first, LT1002/sonepcizumab, which can selectively bind S1P with picomolar affinity and block S1P-induced cytokine release, was terminated due to a lack of efficacy [111]. The second, ABC294640, a selective SphK2 inhibitor, effectively reduced the levels of S1P and inhibited the growth of multiple cancers in vitro and in preclinical xenograft studies [112–114]. This inhibitor is now being studied in a phase I/II clinical trial for DLBCL; the final results are not yet available. In addition, considerable progress has made in preclinical studies focusing on SphK inhibitors (SKIs). Two such SKIs are SKI-I (4-(4′-phenoxyanilino)-6,7-dimethoxyquinazoline) and SKI-II (4-[[4-(4-chlorophenyl)-1,3-thiazol-2-yl]amino]phenol) [115, 116]. By inhibiting SphK activity, these small-molecule compounds reduce S1P levels. The first, SKI-I, is a slightly stronger inhibitor of SPHK1 versus SPHK2 [117] and blocks SPHK1 activity in vitro to decrease JC mammary tumor growth [115]. In mice, by arresting cells in the G2/M and S cell cycle phases and by increasing cell apoptosis, intraperitoneal SKI-I decreases melanoma tumor growth [118]. SKI-II has worse SphK1/SphK2 selectivity compared with SKI-I, although it has been applied extensively in biological investigations of the role of SphK1 and SphK2 in disease and has exhibited impressive anticancer effects [119]. Yang et al. [120] demonstrated that SKI-II more efficiently suppresses the proliferation and survival of two human acute myeloid leukemia cell lines—HL-60 and U937 cells—than two other SphK1 inhibitors (fingolimod and SK1-I). Another study found that SKI-II suppresses the growth of human hepatoma HepG2 cells by decreasing the expression of β-catenin and the downstream molecules in the β-catenin signaling pathway [121]. In addition, SKI-II can also increase the sensitivity of hepatocellular carcinoma cells to the chemotherapeutic drug 5-fluorouracil by inhibiting FAK/IGF-1R and osteopontin signaling [122].

S1P exerts pleiotropic bioactive effects through the S1PRs (S1PR1–5). The roles played by these receptors in cancer are increasingly better understood. Accordingly, one possible cancer therapeutic approach with considerable potential would be to selectively target these receptors with agonists or antagonists. Recently, numerous studies have illustrated the validity of the strategy [67, 93, 123]. As far as S1PR2 is concerned, S1PR2 signaling induces acute myeloid leukemia growth or proliferation [124] and mediates tumor metastasis in bladder cancer and melanoma cells [85]. In addition, S1PR2 can also inhibit tumor angiogenesis in mouse models [125], due to its dual nature in the pathogenesis of cancer. Thus, these data suggest that selective targeting of S1PR2 in cancer cells, at least in acute myeloid leukemia, bladder cancer, and melanoma, may provide an anticancer therapeutic strategy. Unfortunately, however, fingolimod is the only S1PR-related drug used in clinical practice, mainly to treat multiple sclerosis. The reason is that five different S1PR each other and nonselective drug greatly reduce anticancer effect. Furthermore, these wide-ranging anticancer actions may interfere with a number of unrelated signaling pathways, such as SET/PP2A and PI3K/AKT [123] (Table 2).

**Table 2:** Roles played by individual S1PRs in different cancer

S1PRs	Cancers
S1PR1	
Promote invasion and angiogenesis [19, 21, 81] Promote progression [57, 75] Promote migration and invasion [71–73, 76] Promote invasion [77] Promote survival and migration [78–80]	Breast cancer [19, 21, 71–73] Mice B16 tumor [75] Colon cancer [57] Wilms tumor [76] Glioblastoma [77] Fibrosarcoma [79] Hodgkin lymphoma [80]
S1PR2	
Pro-tumorigenesis	
Promote growth and invasion [82]	Cholangiocarcinoma [82]
Promote angiogenesis and growth [11]	Wilms tumor [11]
Promote invasion [83]	Glioma cell U-118 [83]
Promote migration and invasion [84]	Pancreatic cancer [84]
Promote lung metastasis [85]	Bladder cancer [85]
Increase drug resistance [66, 87]	Chronic myeloid leukemia [87]
Anti-tumorigenesis	
Suppress migration [88, 89]	Glioblastoma cells [88, 89]
Suppress invasion [90]	Thyroid cancer C643 cells [90]
Suppress proliferation [12]	Wilms tumor [12]
Lead to diffuse B-cell lymphoma formation [91]	S1PR2 knockout mice [91]
Promote invasion [93]	Repress S1PR2 expression in esophageal squamous cell carcinoma [93]
S1PR3	
Promote progression [95–97]	Breast cancer [95–97]
Promote migration [98]	Nasopharyngeal carcinoma cells [98]
Promote proliferation [102]	Osteosarcoma cells [102]
Promote growth [99–101]	Human lung adenocarcinoma cells [99–101]
S1PR4	
Promote invasion and aggressiveness [105, 106]	Breast cancer cells [105, 106]
S1PR5	
Pro-tumorigenesis	
Promote survival [108]	Prostate cancer cells [108]
Anti-tumorigenesis	
Suppress proliferation and migration [109]	Esophageal squamous cell carcinoma cells [109]

Therefore, a long and arduous road remains, although S1P signaling is nonetheless a promising therapeutic target in cancer. First of all, further research is needed to obtain more detailed information on the S1P signaling pathway. In addition, the development is required of more effective and specific modulators of the various S1P signaling pathway components to maximize therapeutic efficacy and minimize adverse effects such as cytotoxicity.

## Conclusion

In the past two decades, considerable effort has been expended in clarifying the function of S1P signaling in the progression and treatment of cancer, as well as in exploring S1P signaling modulators, such as SphK, S1P transporters and receptors, and S1P agonists and antagonists. S1P and its signaling cascades are strongly linked to numerous aspects of cancer progression, including cell survival, proliferation, and migration, as well as chemotherapy resistance. Given this seemingly hopeless situation, with no effective methods to combat the all-too-often devastating effects of cancer, there is no doubt that further research is warranted to develop targeted therapies. In terms of the existing study results, S1P signaling is likely to be a much-needed novel target for the treatment of cancer. While meeting the opportunity, we should also be prepared to face enormous challenges. (1) Current research results indicate that S1PR1 and S1PR3, which have many similar aspects, both play roles in promoting the occurrence and development of tumors. Therefore, the targeting of S1P1 and S1PR3 may have great therapeutic potential for cancer. However, because the therapeutic efficacy of suppression or gene knockout of S1PR1 or S1PR3 varies widely for different types of tumors (currently only in tumor models or cells), further preclinical and even clinical trials are necessary. (2) S1PR2 is a double-edged sword, which has the dual role of promoting cancer and inhibiting cancer. Most of the existing studies only indicate the role of S1PR2 in certain tumors, and the next breakthrough involving S1PR2 will probably rely on actively exploring this dual-effect mechanism in order to reduce or remove the cancer-promoting effect and/or improve the anti-cancer effect. (3) Compared with S1PR1–3, S1PR4 and S1PR5 have a restricted distribution and less clear functions, and further research is needed to obtain more detailed information on S1P4 and S1PR5. In addition, there are close and complex interactions among S1P, SphK, S1P transporter, its degrading enzymes, and S1PR1–5, and they all play important roles in cancer, inflammation, immune, and angiogenesis. Elucidation of these complex interactions may be one of the most demanding future challenges. In addition, recent efforts have produced a variety of S1P agonists/antagonists that target different S1PRs, some of which have been shown to have major roles in cancer. For instance, FTY720, an S1P agonist that binds to S1PR1, S1PR3, S1PR4, and S1PR5, has been indicated to suppress the growth and aggressiveness of tumor in several cancer models. In addition, an S1P antagonist, CYM-5478, may promote tumor aggressiveness by targeting S1PR2 in oral squamous cell carcinoma. However, other S1P agonists and antagonists have not been researched or tested in cancer models. Therefore, more studies are needed to evaluate the effects of these S1P agonists/antagonists in cancer. With the increased focus on S1P agonists/antagonists in cancer and the resultant progress, S1P agonists/antagonists might become a promising therapy for cancer patients.

## Data Availability

Not applicable.

## Abbreviations

S1P: sphingosine-1-phosphate
SphKs: sphingosine kinases
ABCC1: ATP-binding cassette C1
ABCG2: ATP-binding cassette G2
Spns2: spinster 2
S1PRs: S1P receptors
Gq, Gi, and G12/13: G protein α subtypes
AKT: protein kinase B
ERK1/2: extracellular regulated protein kinases
SphK1: sphingosine kinase 1
SphK2: sphingosine kinase 2
GPCRs: G protein-coupled receptors
LPA: lysophosphatidic acid
CXCR4: chemokine receptor type 4
Ras: rat sarcoma viral oncogene
ERK: extracellular regulated protein kinases
NLRP3: NACHT, LRR and PYD domains-containing protein 3
IL-1β: interleukin-1β
PI3K: phosphoinositide 3-kinase
STAT3: signal transducer and activator of transcription 3
PLC: phospholipases C
uPA: urokinase plasminogen activator
KLF2: Krüppel-like factor 2
COX-2: cyclooxygenase 2
MMP-9: matrix metalloproteinase-9
NF-κB: nuclear factor kappa-light-chain-enhancer of activated B cells
CTGF: connective tissue growth factor
YAP: Yes-associated protein
ROS: reactive oxygen species
ROCK: Rho-associated kinase
JNK: c-Jun N-terminal kinases
DLBCL: diffuse large B cell lymphoma
TGF-β: transforming growth factor beta
TGF-βR2: transforming growth factor β receptor 2
SMAD1: Mothers against decapentaplegic homolog 1
SMAD3: Mothers against decapentaplegic homolog 3
EGFR: endothelial growth factor receptors
EGF: epidermal growth factor
c-MYC: cellular-myelocytomatosis viral oncogene
PGAM1: phosphoglycerate mutase 1
HER2: human epidermal growth factor receptor-2
SKIs: SphK inhibitors
PP2A: protein phosphatase 2
SET: patient se translocation
